# Usage of computational method for hemodynamic analysis of intracranial aneurysm rupture risk in different geometrical aspects

**DOI:** 10.1038/s41598-023-48246-7

**Published:** 2023-11-25

**Authors:** Mehdi Fattahi, Seyyed Amirreza Abdollahi, Ali Hosin Alibak, Saleh Hosseini, Phuyen Dang

**Affiliations:** 1 Institute of Research and Development, Duy Tan University, Da Nang, Vietnam; 2 School of Engineering and Technology, Duy Tan University, Da Nang, Vietnam; 3 Faculty of Mechanical Engineering, University of Tabriz, Tabriz, Iran; 4 Petroleum Engineering Department, Faculty of Engineering, Soran University, Soran, Kurdistan Region, 44008 Iraq; 5 Department of Chemical Engineering, University of Larestan, Larestan, Iran

**Keywords:** Biomedical engineering, Mechanical engineering

## Abstract

The importance of the parent vessel geometrical feature on the risk of cerebral aneurysm rupture is unavoidable. This study presents inclusive details on the hemodynamics of Internal carotid artery (ICA) aneurysms with different parent vessel mean diameters. Different aspects of blood hemodynamics are compared to find a reasonable connection between parent vessel mean diameter and significant hemodynamic factors of wall shear stress (WSS), oscillatory shear index (OSI), and pressure distribution. To access hemodynamic data, computational fluid dynamics is used to model the blood stream inside the cerebral aneurysms. A hemodynamic comparison of the selected cerebral aneurysm shows that the minimum WSS is reduced by about 71% as the parent vessel’s mean diameter is increased from 3.18 to 4.48 mm.

## Introduction

Endovascular coiling is a minimally invasive technique used to treat intracranial aneurysms and reduce the risk of rupture. It involves inserting small metal coils into the aneurysm sac, promoting thrombosis (blood clotting) and reducing blood flow into the aneurysm. The coiling procedure has several effects on the hemodynamics of the aneurysm, contributing to the reduction in rupture risk. Here are some ways in which endovascular coiling influences hemodynamics and reduces aneurysm rupture risk^1–3^.

Coiling disrupts the normal blood flow patterns within the aneurysm by filling the sac with coils. This promotes flow diversion away from the aneurysm, reducing the impact of high-velocity jets and flow impingement on the vulnerable aneurysm wall. By redirecting blood flow, coiling helps alleviate the hemodynamic stress that can lead to aneurysm rupture^4–6^.

The coils placed within the aneurysm initiate thrombosis, leading to the formation of a blood clot. As the clot matures and organizes, it further reduces blood flow into the aneurysm, effectively occluding the sac. The presence of the clot and subsequent endothelialization (formation of a new endothelial layer) over the coils promotes stabilization of the aneurysm, reducing the risk of rupture^7–9^.

Coiling alters the flow dynamics within the aneurysm, resulting in a reduction in wall shear stress (WSS). High WSS has been associated with aneurysm growth and rupture. By disrupting the flow patterns and reducing the forces acting on the aneurysm wall, coiling helps decrease the WSS, which can contribute to the long-term stability of the aneurysm^10–13^.

The presence of coils within the aneurysm initiates a healing response in the vessel wall. Over time, this can lead to positive remodeling, where the vessel wall thickens and strengthens around the coils. Positive remodeling reinforces the weakened aneurysm wall, enhancing its resistance to rupture. The presence of coils within the aneurysm provides a physical barrier that prevents the circulation of emboli (blood clots) from entering the cerebral circulation. This reduces the risk of thromboembolic events, which can further contribute to aneurysm rupture. Endovascular coiling, by altering the hemodynamics of the aneurysm, promotes flow diversion, aneurysm occlusion, and positive vessel remodeling^14–16^. These changes collectively contribute to the reduction in aneurysm rupture risk. However, it's important to note that the effectiveness of coiling can vary depending on factors such as aneurysm size, shape, and location. Patient-specific evaluation and careful consideration of the hemodynamic impact are crucial for successful treatment outcomes^17–20^.

Endovascular coiling is a widely used treatment technique for intracranial aneurysms^21, 22^. While it can be effective for many aneurysms, the suitability of coiling as a treatment option depends on several factors, including the size and shape of the aneurysm. Endovascular coiling is commonly used for small to medium-sized aneurysms. Small aneurysms, typically defined as those with a maximum diameter less than 5 mm, are often ideal candidates for coiling due to their favorable outcomes and lower risk of rupture. Medium-sized aneurysms (5–15 mm) can also be treated with coiling, although the decision may depend on other factors such as the location and morphology of the aneurysm. Aneurysms come in various shapes, including saccular, fusiform, and irregular^23–26^. Saccular aneurysms with a well-defined neck and dome are generally more amenable to coiling. The presence of a narrow neck allows for better coil placement and stability within the aneurysm. Fusiform and irregular-shaped aneurysms, on the other hand, may present challenges for coiling due to their complex geometry and involvement of elongated segments. In such cases, alternative treatment options, such as flow diverters or surgical clipping, may be considered. The location of the aneurysm can also influence the feasibility of coiling. Aneurysms located in the anterior circulation, such as the anterior communicating artery or middle cerebral artery, are more commonly treated with coiling due to better accessibility and favorable outcomes. However, aneurysms in challenging locations, such as the posterior circulation or complex bifurcations, may pose technical difficulties for coiling^27–29^.

It's important to note that the decision regarding the most appropriate treatment approach for an aneurysm is made on an individual basis, considering multiple factors such as aneurysm size, shape, location, patient's overall health, and the expertise of the treating physician^30–33^. In some cases, a combination of treatment techniques may be employed, such as using a flow diverter in conjunction with coiling or opting for surgical clipping for complex aneurysms. A multidisciplinary team, including neurosurgeons, interventional neuroradiologists, and neurologists, collaboratively assess each case to determine the most suitable treatment strategy for the patient's specific circumstances^34–37^.

There are several scientific articles that present the hemodynamic analysis of the cerebral analysis via a computational approach. Unlike previous papers^38–41^, in this paper, comprehensive investigations on the hemodynamics of the ICA aneurysm have been performed to disclose the effects of parent vessel mean diameter on the risk of aneurysm bleeding. The hemodynamic factors i.e. wall shear stress (WSS), oscillatory shear index (OSI), and pressure distribution on three different ICA cases have been developed and analyzed by computational fluid dynamic method. In addition, the influence of the endovascular coiling of the aneurysm on the blood flow characteristic is also revealed in the selected aneurysm.

## Aneurysm selection and computational technique

For the selection of the aneurysm geometry, several rupture/un-rupture cerebral aneurysms have been obtained from the Aneurisk website^42^ which contains geometrical features of the different patients from Emory University. It is confirming that all methods were carried out in accordance with relevant guidelines and regulations. Besides, all experimental protocols were approved by of the Ca' Granda Niguarda Hospital and it is confirmed that informed consent was obtained from all subjects and/or their legal guardian(s).

**Figure 1:**
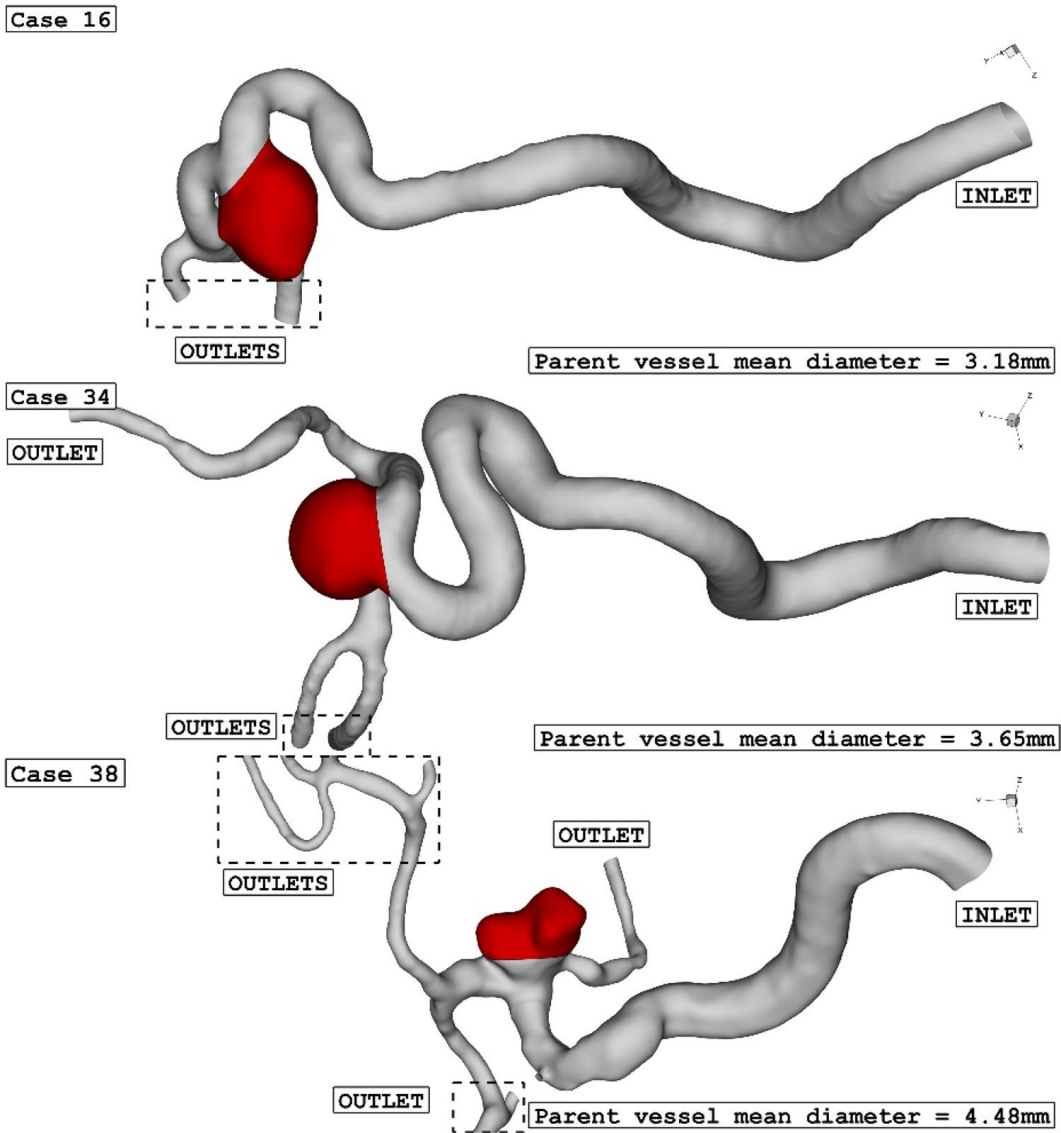
ICA aneurysm geometry of 3 different cases.

The three selected ICA aneurysms have different parent vessel mean diameters as presented in Table 1. As offered in Table 1, the size of the Parent vessel mean Diameter of the chosen cases is varied between 3.18 mm and 4.48 mm, and all cases are related to female patients. Table 1 also presents the HCT of the selected female patient and the sac section area. The size of the latter is in a limited range of (47–54 mm^2^). Figure 1 displays the chosen saccular aneurysm with geometrical details.

**Table 1:** Geometry details of selected aneurysms.

Case ID	Parent vessle mean diameter (mm)	Sac section area (mm^2^)	Sex
16	3.18	47	Female (HCT = 0.40)
34	3.65	54	Female (HCT = 0.40)
38	4.48	50	Female (HCT = 0.40)

**Figure 2:**
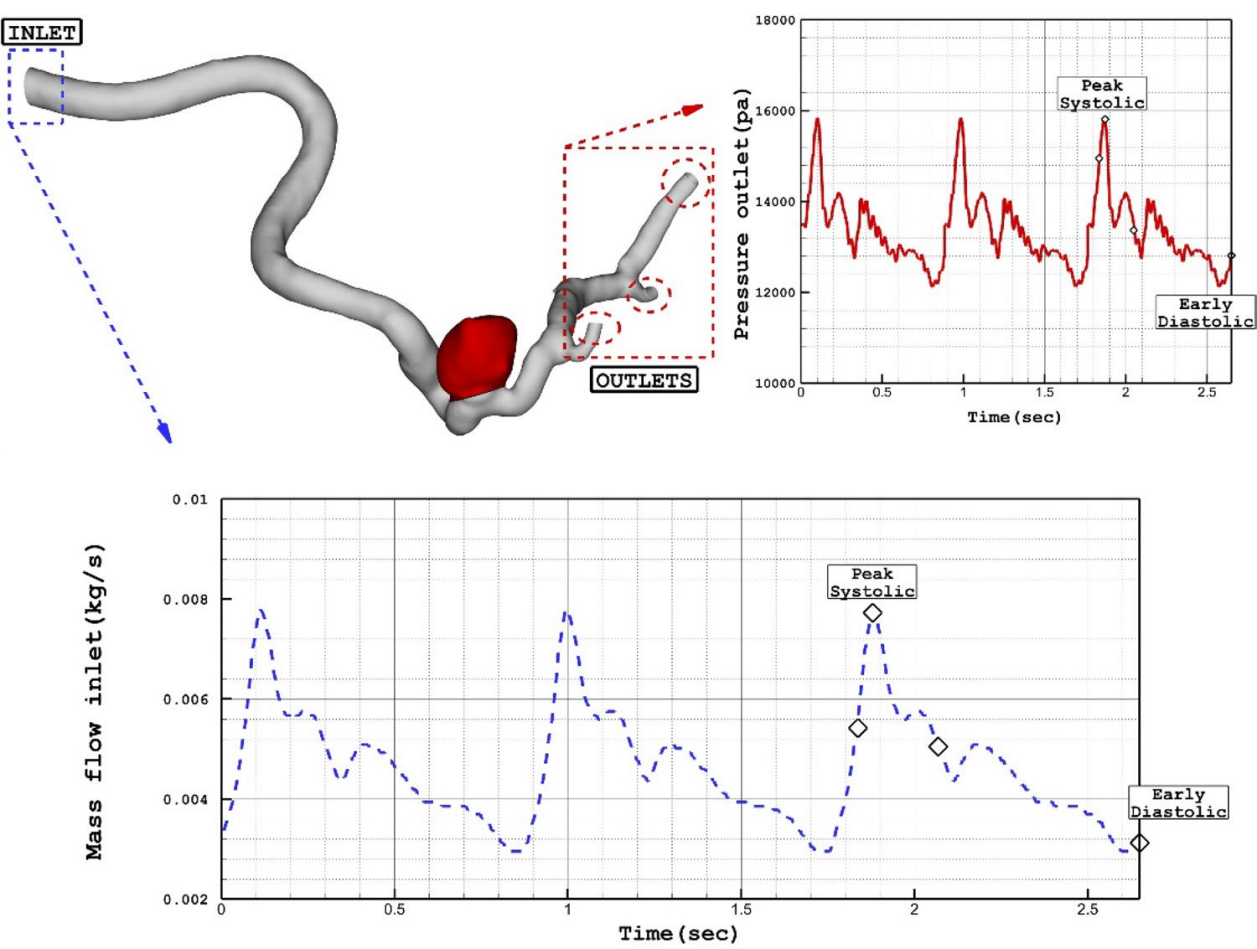
Applied mass and pressure profile at inlet and outlets.

The simulation of the blood flow is done via solving transient Navier–stocks equations while it is assumed that the blood flow is not Newtonian and the one-way FSI model is considered for the interactions of the vessel with the incoming blood stream^37^. Casson model is applied for the estimation of blood viscosity in our study^38, 39^ and blood flow is considered laminar^40^. The applied boundary condition at the outlet and inlet is also displayed in Fig. 2. Two significant stages of peak systolic and early diastolic are also displayed in Fig. 2. The results of OSI are calculated.at the end of 3rd cardiac cycle of early diastolic as demonstrated in Fig. 2. The influence of the coiling is investigated by applying a porous domain in the sac section area with a porosity value of 0.844 as presented in Table 2.

**Table 2:** Porosity of coiled aneurysms.

HCT	Porosity	Viscous resistance (m2/1)
0.4	0.844	16,958,264.02

**Figure 3:**
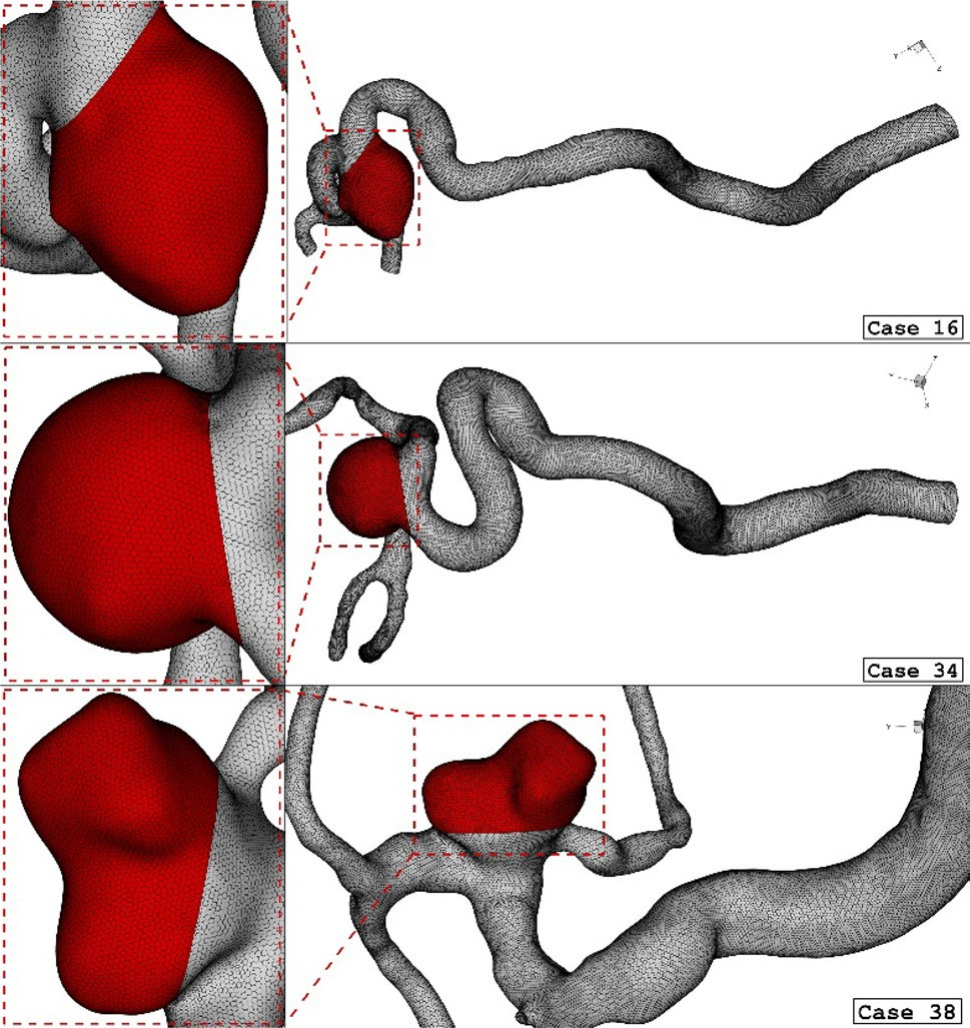
Grid generation for 3 different ICA cases.

The generated grid for the simulation of the blood flow on the selected cerebral aneurysm is displayed in Fig. 3. Efficient grid for numerical study is essential step^43–47^. The size of the grid on the vessel is almost uniform and the resolution of the produced grid on the cross-section of the parent vessel is higher in the vicinity of the aneurysm and vessel wall. As demonstrated in Fig. 3, the sac section region is split for applying the coiling porosity which is explained before. A close-up view is presented for the sac section area in the presented figure. To ensure the grid resolution, more than five grid sizes are produced and the average WSS on the sac section area is calculated. Finally, the range of grid cells for the selected cases is within 1.1 million cells and 1.6 million cells. The using computational methodology for resolving real scientific problem is efficient and time consuming^48–50^.

## Results and conclusion

The details of hemodynamic factors related to the simulation of the aneurysms are presented in Table 3. In this table, the values of mean WSS, minimum WSS, OSI, wall pressure, and average velocity of the blood flow are reported. Except for OSI, all other factors are calculated for the peak systolic stage. In the following, the physical aspects of these achieved data are fully explained with a graphical contour.

**Table 3:** Results of hemodynamic factors.

	Parent vessel mean diameter (mm)	Sac section area (mm^2^)	WSS_mean (Pa)	OSI_mean	Wall pressure_mean (Pa)	Aneurysm velocity_mean (m/s)
Case 16	3.18	47	9.368811	0.03234751	19,707.41	0.4137481
Case 34	3.65	54	8.146665	0.02603191	25,071.71	0.4034861
Case 38	4.48	50	2.778162	0.01463711	19,287.28	0.1811908

**Figure 4:**
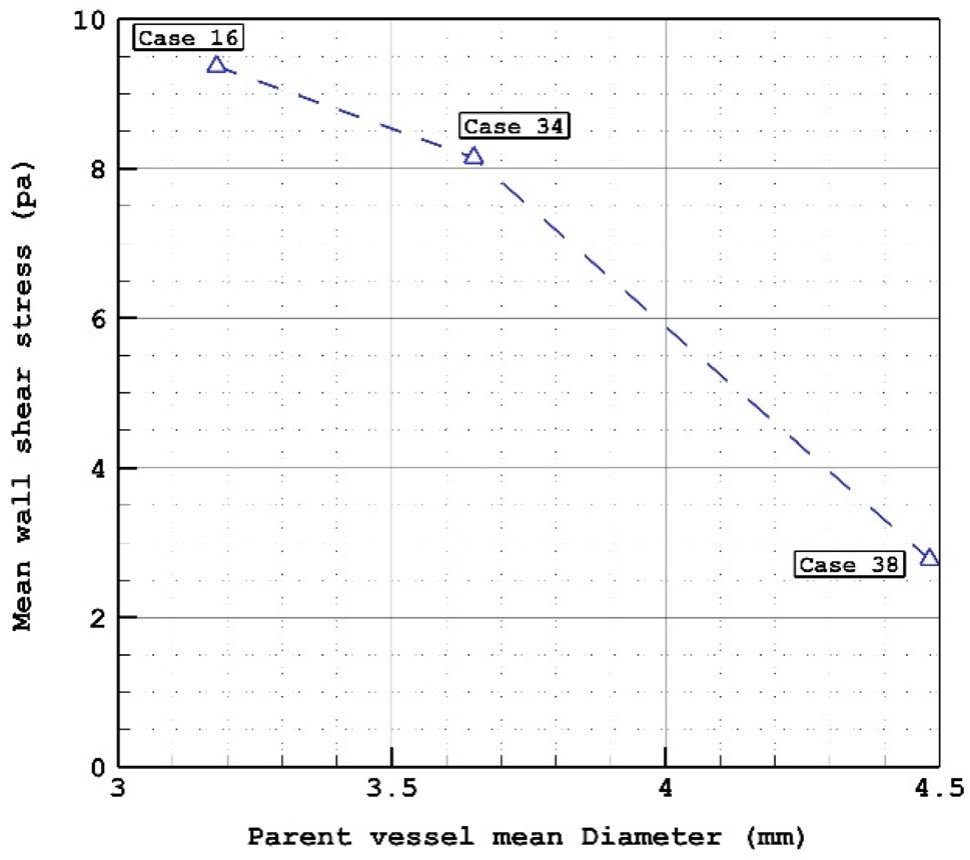
Mean wall shear stress versus parent vessel mean diameter at peak systolic.

**Figure 5:**
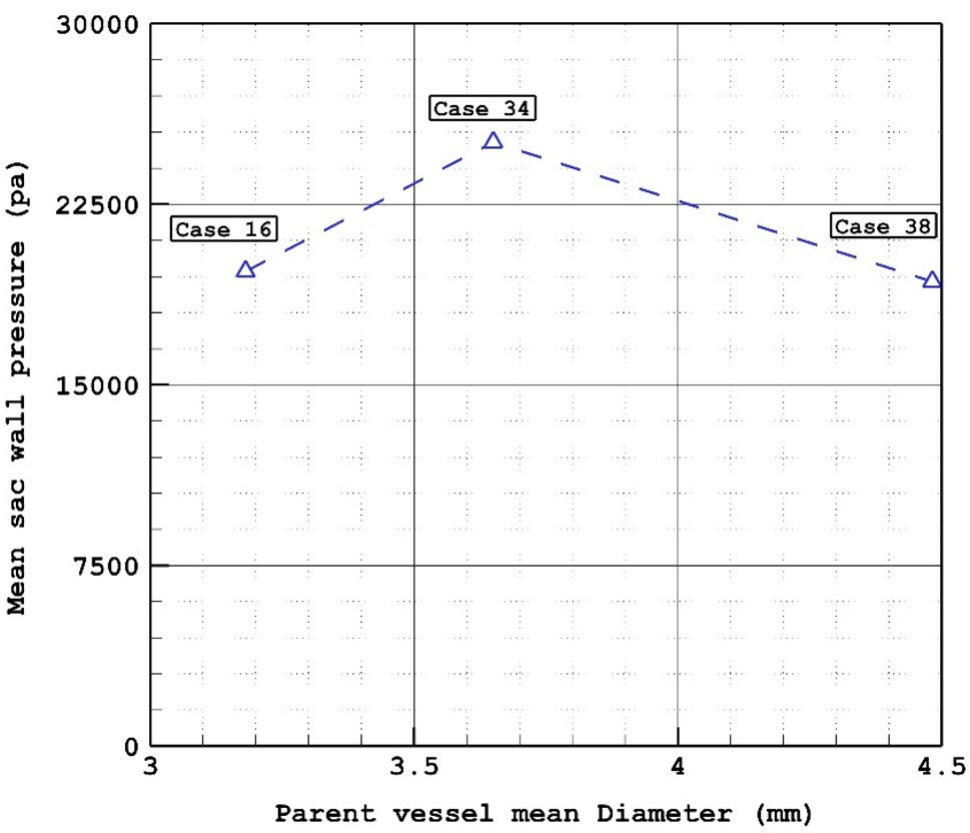
Mean wall pressure versus Parent vessel mean diameter at peak systolic.

The effects of the parent vessel's mean diameter on the mean WSS are demonstrated in Fig. 4. As plotted in Fig. 4, increasing parent vessel mean diameter considerably decreases the minimum WSS on sac surface. 80% reduction in mean WSS is attained at peak systolic when parent vessel mean diameter is increased about 40%. To recognize the effects of parent vessel mean diameter, Fig. 5 displays the WSS contour on sac area at peak systolic. Evaluation of WSS distribution indicates that the most critical area on the sac surface is neck section. In fact, in the neck section, the blood flow enters the sac and exit from sac with limited cross section area. The close-up view presents more details about the critical section in the neck section.

**Figure 6:**
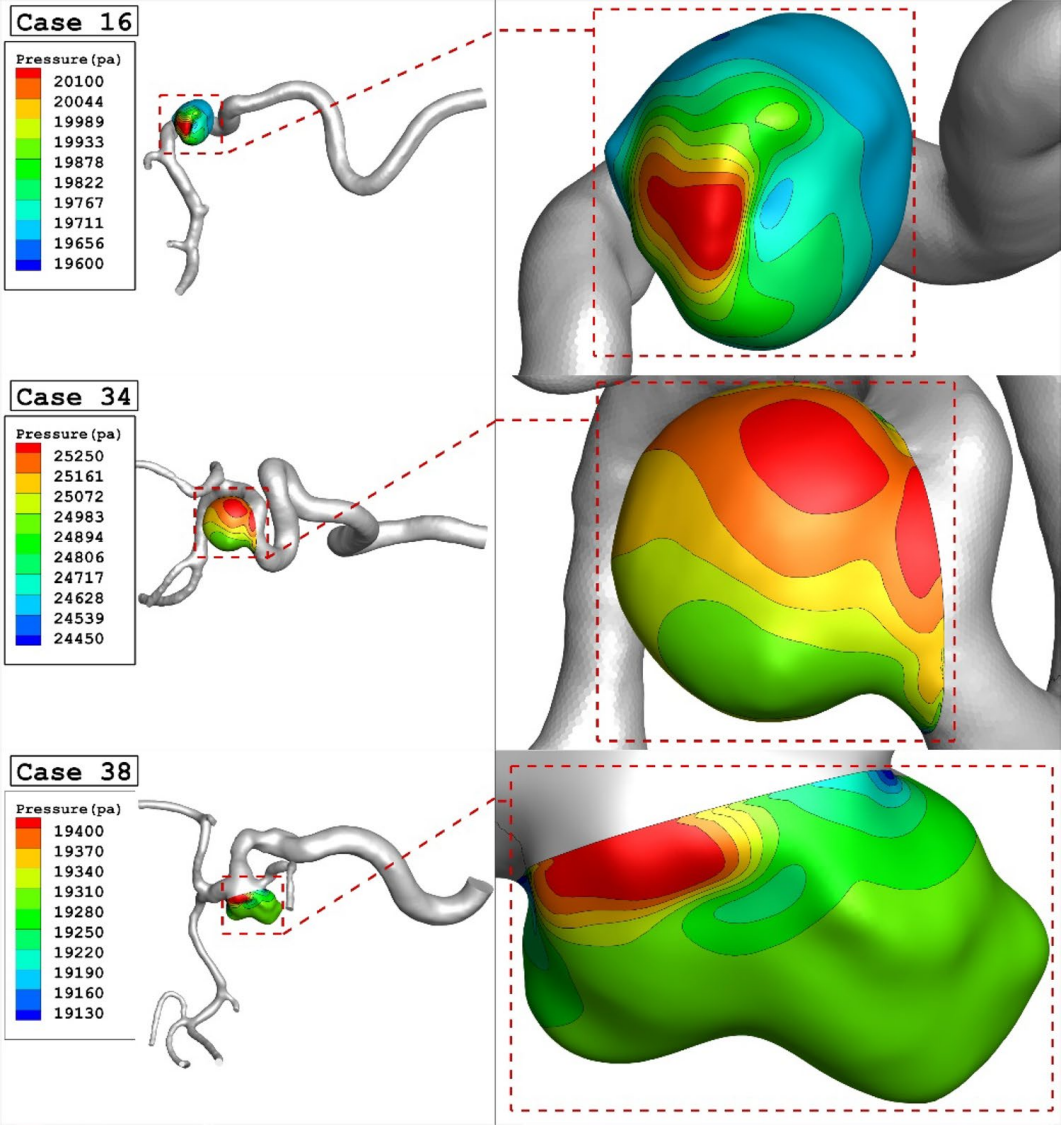
Wall pressure contours (Peak systolic) in different cases.

The variation of the mean sac wall pressure under impacts of different parent mean vessel are exhibited in Fig. 5. The figure shows that there is not a meaningful connection between the mean sac wall pressure and parent vessel mean diameter at peak systolic stage. Although, the change of parent vessel mean diameter in the chosen patients are high, the mean sac wall pressures varies restrictively. Figure 6 displays the changes of pressure contour on the sac surface in chosen models. In case 16, the maximum pressure is noticed in dome section while critical high pressure region happens near neck region in case 34. In case 38, neck region is critical from pressure aspects.

**Figure 7:**
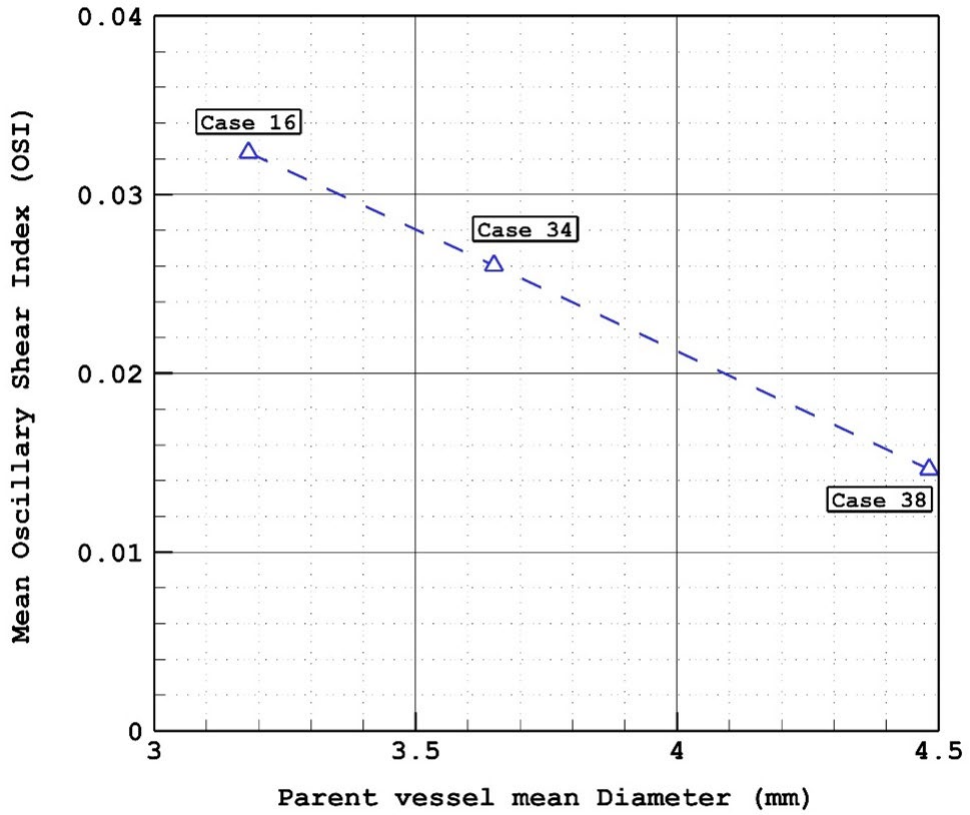
Mean OSI versus Parent vessel mean diameter at early diastolic.

**Figure 8:**
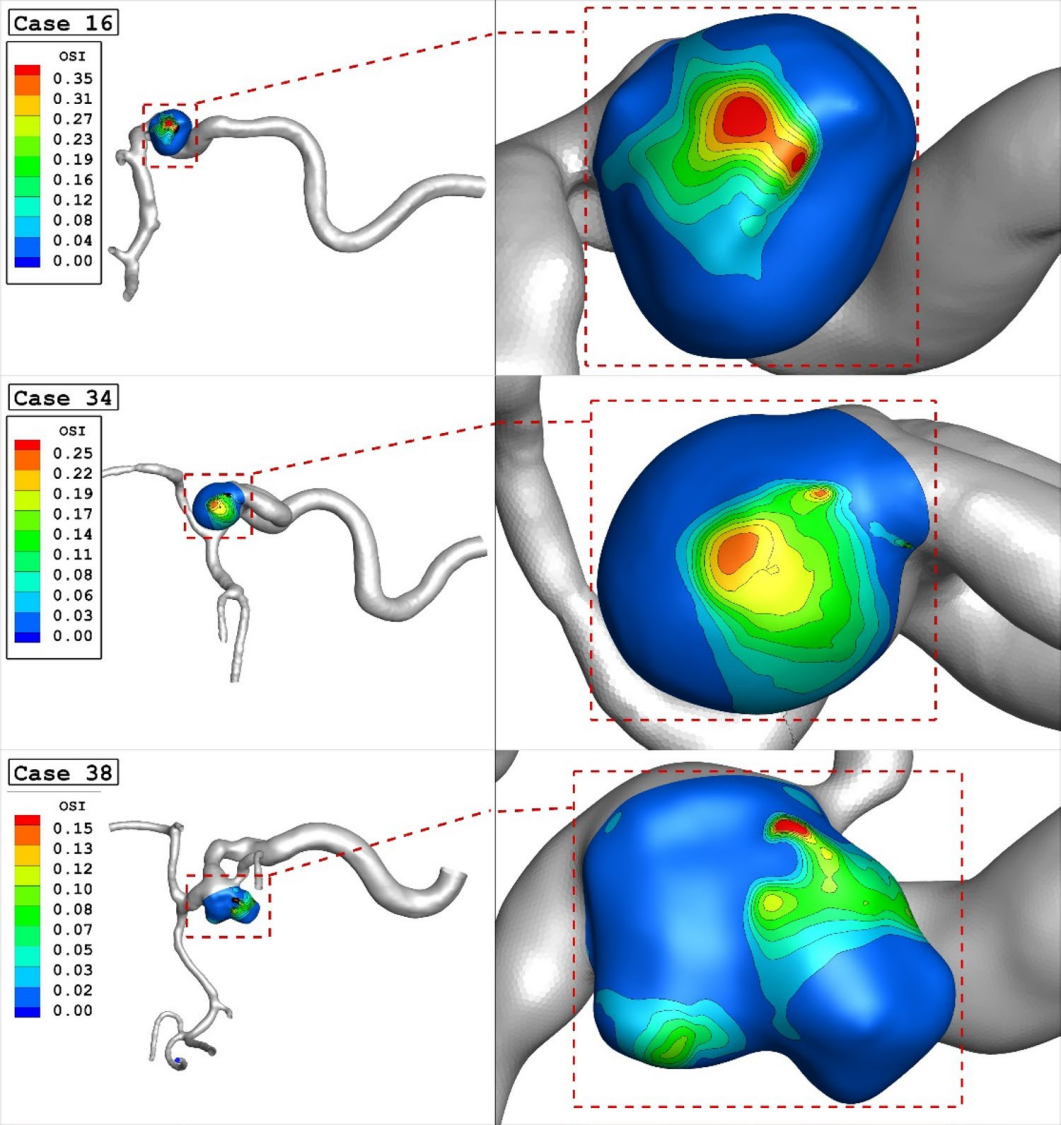
OSI contours (Early diastolic) in different cases.

The changes of mean OSI for different parent vessel mean diameters are displayed in Fig. 7. The results show that increasing parent vessel mean diameter about 40% would decrease the mean OSI on the sac area about 43% at early diastolic. As demonstrated in Fig. 8, the most critical region for the rupture from OSI aspects is dome region where has higher OSI value. It is also found that the maximum OSI value on the dome area is also reduced from 0.35 to 0.15 by increasing the diameter of the parent vessel.

**Figure 9:**
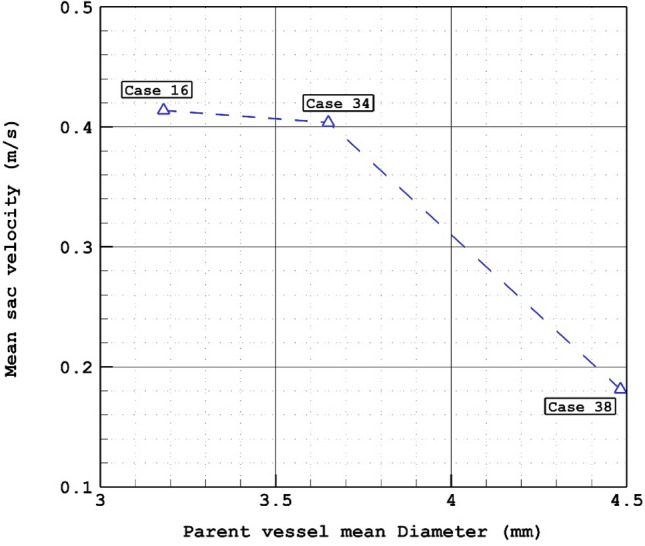
Mean sac velocity versus Parent vessel mean diameter at peak systolic.

**Figure 10:**
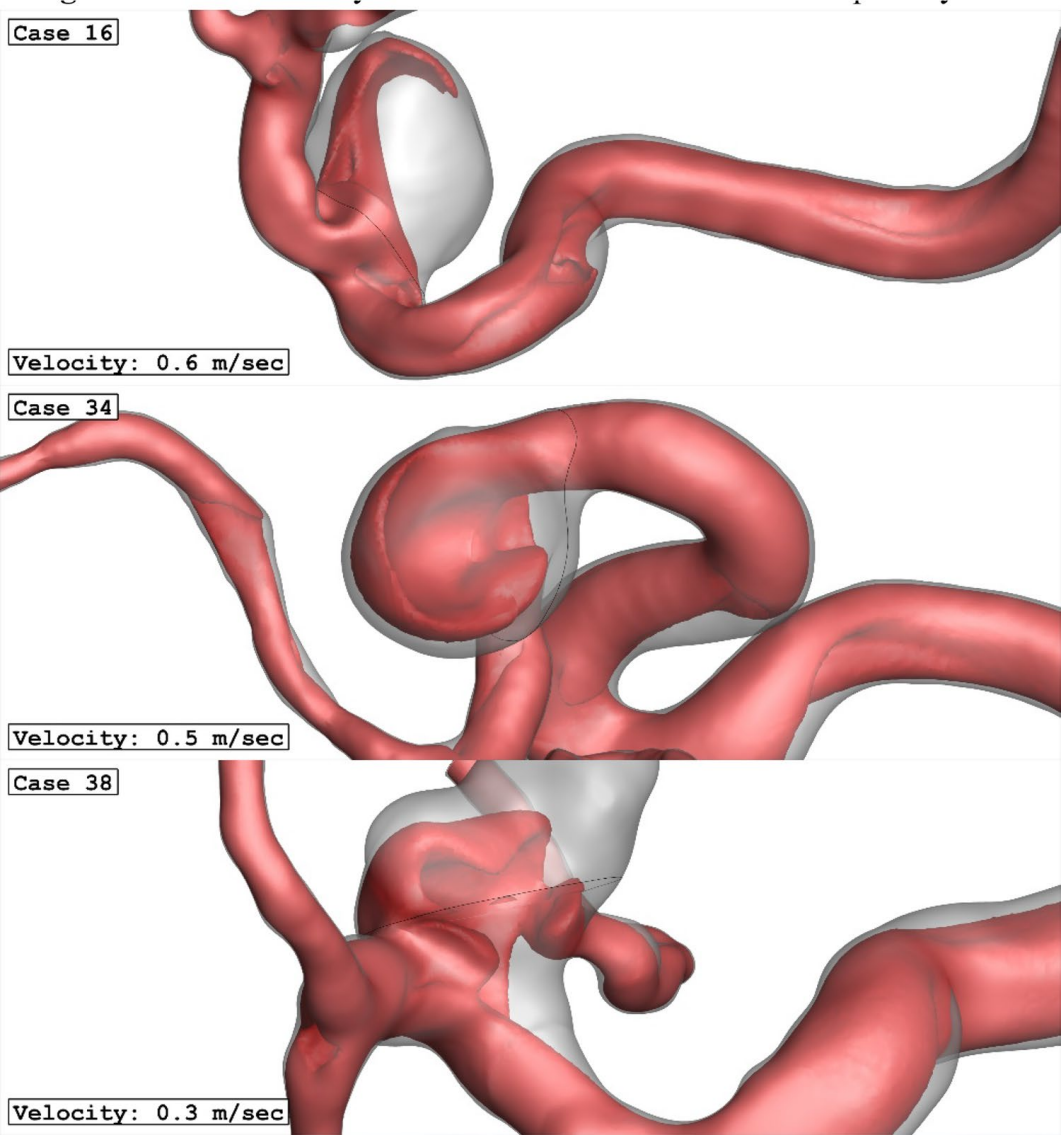
Iso-Surface (velocity at peak systolic) in different cases.

Figure 9 illustrates the impacts of parent vessel mean diameter on the mean velocity inside the sac region. As demonstrated in Fig. 10, 44% reduction in mean velocity of the blood velocity is noticed as 41% increase in parent vessel mean diameter is observed. The structure of the blood flow inside the cerebral aneurysm is compared for the selected patients by demonstrating the iso-velocity contour in Fig. 10. The structure of the iso-velocity indicates that the velocity in center of the aneurysm is less than other region and the shape of aneurysm is very important on the feature of blood flow.

## Conclusion

The present study has focused on the impacts of the parent vessel mean diameter on the hemodynamic aspects of the cerebral aneurysm with endovascular coiling. Computational approach is used to model the transient blood flow inside the parent vessel and sac area while it is presumed that the blood is not Newtonian fluid. The contours of pressure, WSS and OSI of three nominated ICA aneurysms are compared to disclose the importance of the parent vessel mean diameter on the probability of aneurysm bleeding. Based on the OSI results at early diastolic, increasing parent vessel mean diameter about 40% would decrease the mean OSI on the sac area about 43%.

## Data Availability

All data generated or analysed during this study are included in this published article.
